# Effects of Baduanjin Exercise on Physical Function and Health-Related Quality of Life in Peritoneal Dialysis Patients: A Randomized Trial

**DOI:** 10.3389/fmed.2021.789521

**Published:** 2021-11-29

**Authors:** Fan Zhang, Jing Liao, Weihong Zhang, Hui Wang, Liuyan Huang, Qiyun Shen, Huachun Zhang

**Affiliations:** Longhua Hospital, Shanghai University of Traditional Chinese Medicine, Shanghai, China

**Keywords:** Baduanjin, exercise, peritoneal dialysis, physical function, quality of life

## Abstract

**Background and Aims:** Exercise is an efficient non-pharmacological intervention for chronic kidney disease. The study aims to evaluate the effects of Baduanjin exercise on physical function and health-related quality of life (HRQOL) in peritoneal dialysis (PD) patients.

**Methods:** Seventy PD patients were randomly assigned to either the Baduanjin exercise group or the control group. Fifty-seven patients completed the study (exercise group, 25; control group, 32). The exercise group received the Baduanjin exercise program for 12 weeks. The control group received usual care. Three well-established performance-based tests determined physical function: five times sit-to-stand test (FTSST), timed up and go test (TUGT), and handgrip strength (HGS). HRQOL was assessed by the Kidney Disease Quality of Life-Short Form.

**Results:** At baseline, no differences in physical function and HRQOL were observed between the Baduanjin exercise and the control group. At follow-up, the Baduanjin exercise group showed a marginally significant improvement in FTSST (*P* = 0.008) and TUGT (*P* = 0.040) over the 12 weeks compared to the control group. HRQOL in the Baduanjin exercise group was significantly higher than that of the control group.

**Conclusions:** A 12-week Baduanjin exercise program may improve physical function and HRQOL in PD patients. Longer follow-up is needed to determine if these findings will translate into clinical application.

## Introduction

Chronic kidney disease (CKD) is a growing public health concern in China due to its high prevalence with an incidence of ~10.8% in adults (1), association with increased morbidity, mortality, and progression to end-stage renal disease (ESRD) (2). Peritoneal dialysis (PD) is one of the renal replacement therapies for patients with ESRD. The estimated prevalence of PD was 39.95 per million population in China, and the corresponding number of PD patients was ~55,000 (3).

PD patients generally engage in a low level of physical activity compared to healthy individuals, reducing exercise capacity, decreasing anabolic stimuli, and compromising muscle endurance, muscle strength, and cardiopulmonary fitness (4). Meanwhile, physical inactivity is an independent risk factor for lower health-related quality of life (HRQOL) and higher mortality in PD patients (5). Therefore, most researchers reported exercise as one of the best non-pharmacologic therapies to treat CKD (6), as it may have a protective role concerning residual renal function (7).

PD treatment may imply some challenging and specific physical factors. Whether to exercise with fluid in the abdomen or without is still a controversial issue (6). In general, PD patients should perform moderate-to-high intensity exercise when there is no dialysis fluid in the abdominal cavity, while low-intensity exercise (e.g., walking) can be performed while dialysis fluid left (8). In addition, added weight, hernias, dialysate leakage, and uncertainty exercise prescription limited participation in exercise programs in PD patients due to little known about it (9).

As one of the traditional Chinese exercises, Baduanjin is an aerobic mind-body exercise with low-to-moderate intensity physical activity that is safe for non-communicable diseases and older individuals (10). At present, it has been confirmed that the incorporation of Baduanjin exercise into the lifestyle can significantly improve pulmonary function in patients with the chronic obstructive pulmonary disease (11), glycemic control in patients with diabetes (12), fatigue in patients with heart failure (13), and insomnia symptoms (14). However, what is not yet clear is the impact of Baduanjin on PD patients.

To fill the knowledge gap, the purpose of this study is to evaluate the effectiveness of a Baduanjin exercise program on physical function and HRQOL in PD patients.

## Materials and Methods

### Study Population

Participants were identified from the Department of Nephrology, Longhua Hospital Shanghai University Traditional Chinese Medicine. They were included if they were 18 years or older, were diagnosed as CKD according to the Nation Kidney Foundation-Kidney Disease Outcomes Quality Initiative guidelines (15), and received PD therapy for more than 3 months, and patients were followed regularly at the outpatient clinic every 2 weeks unless loss to follow-up.

Patients were excluded if they needed crutches to walk, had unstable medical conditions (e.g., uncontrolled hypertension: blood pressure >160/100 mmHg, cardiovascular disease (e.g., congestive heart failure)), had participated in similar exercise intervention within the prior 6 months.

### Dialysis Protocol

All patients were treated with glucose-based dialysis fluid for continuous ambulatory peritoneal dialysis containing a 1.5 mmol/L or 2.5 mmol/L concentration. The dialysis frequency was 3–4 times/d, and each abdominal retention time was 3–5 h. Daily dialysate volume was 6–8 L per day.

### Study Procedure

**Figure 1:**
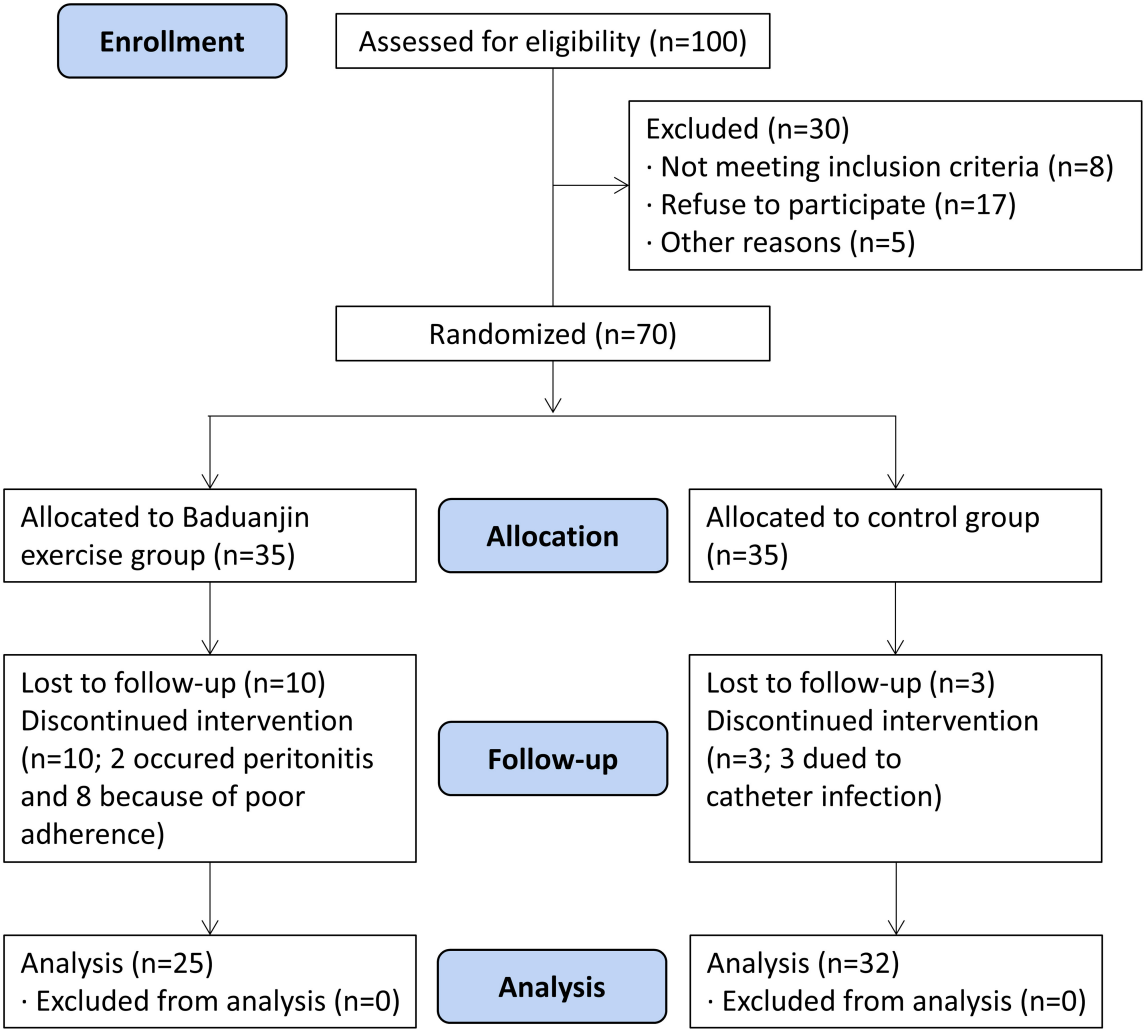
Flow of patients through the study. Of 100 patients who were screened, 70 patients were considered eligible and were randomly assigned to the Baduanjin exercise group or control group. In the exercise group, 25 patients completed the study, compared with 32 patients in the control group.

The study was proved by the Ethics Committee of Longhua Hospital Shanghai University Traditional Chinese Medicine. All patients included in this study signed informed consent. Patients were randomly assigned to two groups of Baduanjin exercise group and control group by a researcher using a computer-generated table of random numbers (random seed: 20190101). The study recruitment process is outlined in Figure 1.

### Control Group

As usual care, nurses provided PD patients with usual care (e.g., conventional medication, routine health guidance) and management strategies for PD-related complications at each outpatient visit.

### Baduanjin Exercise Group

**Figure 2:**
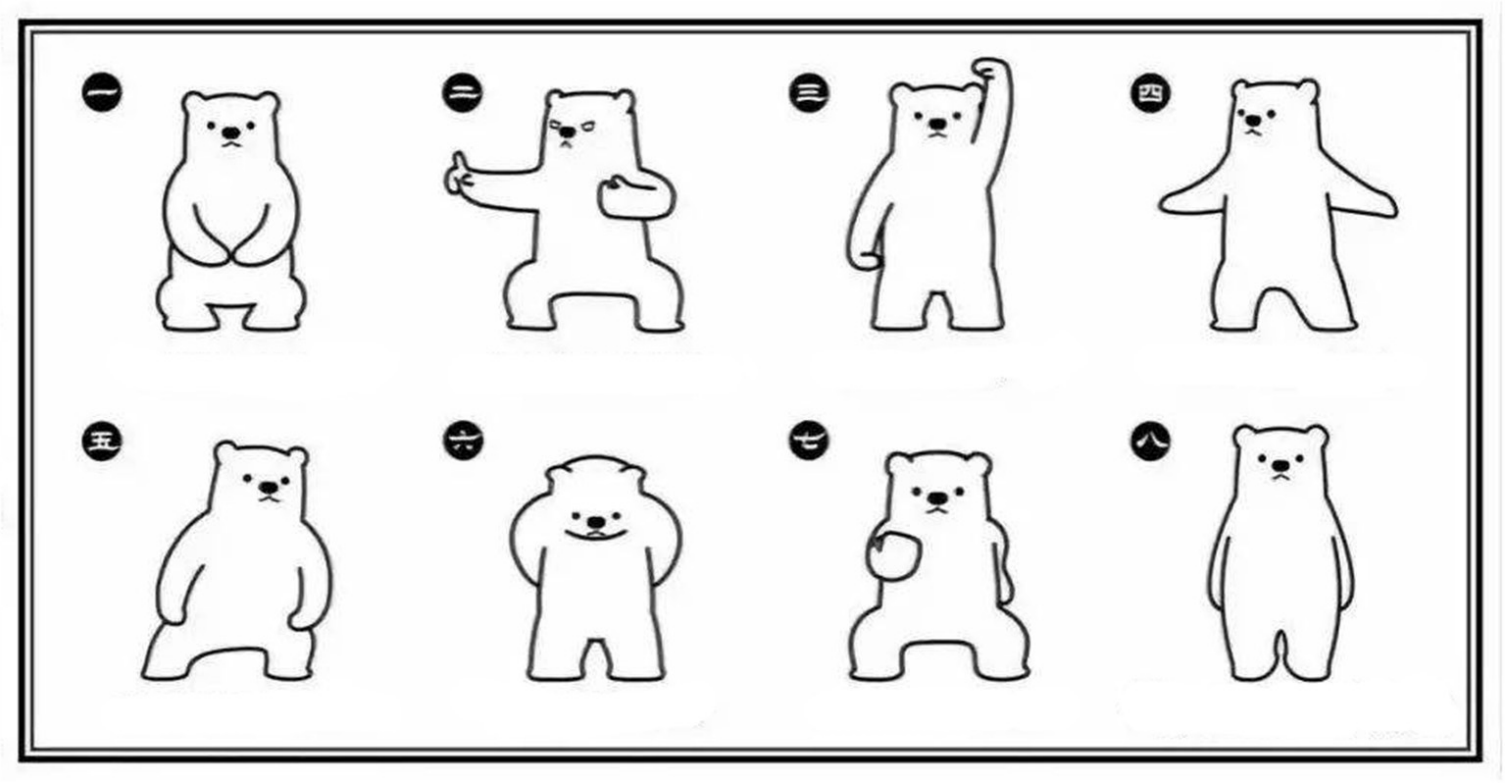
Graphic of Baduanjin. (Extracted from “Wu Shu Shi Jie Pin Dao”, a WeChat Official Account).

Participants allocated to the exercise group received a 12-week home-based Baduanjin exercise program. Before the Baduanjin exercise program, three nurses (LJ, ZHW, and WH) received professional training to provide Baduanjin instruction to the participants. The Baduanjin exercise program includes eight forms (Figure 2): form 1, propping un the sky; form 2, drawing the bow; form 3, raising one hand; form 4, looking over the shoulders; form 5, clenching fists and looking forward with eyes wide open; form 6, pulling the toes; form 7, swaying head and buttocks; form 8, jolting, and about 30 min each time. The duration of intervention was five times a week for 12 weeks, and supervision was provided via *WeChat* message by the trained researchers (LJ, ZHW, and WH). Because Baduanjin is a low-intensity aerobic exercise, patients can perform it while the dialysis fluid is retained in the abdomen (8).

### Primary Outcome

Three well-established performance-based tests were used to assess changes in physical function (16). The five times sit-to-stand test (FTSST) measured lower-extremity muscle strength as a function of the time patients needed to stand up from a seated position and sit back down five times from a chair of standardized height. The timed up and go test (TUGT) was used to measure functional mobility that the time needed to stand up from a chair, walk 3 m, and return to the chair and sit down. The handgrip strength (HGS) was used to measure forelimb muscle strength with a dynamograph.

### Secondary Outcome

The Kidney Disease Quality of Life-Short Form (KDQOL-SF) scale version 1.3 assessed HRQOL in PD patients (17). The scale included 36 items in five dimensions: physical component score, mental component score, burden of kidney disease, symptom/problem, and effect of kidney disease. According to published guidelines, we calculated and linearly converted KDQOL scores to a 0- to 100-point scale, with higher scores reflecting better HRQOL (18). The Cronbach's alpha and test-retest reliability for the Chinese version of the KDQOL-SF was 0.69–0.78 and 0.70–0.86, respectively (19).

### Data Analysis

Data were analyzed using SPSS statistics version 21.0 (IBM Corporation, Armonk, NY, USA). Continuous data were assessed for normality using the Shapiro-Wilk normality test. Data conformance to the normal distribution is described by mean ± standard deviation (x ± SD), and the *t*-test was used to compare the data. Otherwise, non-normal distribution data were expressed as median (quartile range) and were compared using the Mann-Whitney *U* test. The counting data were expressed as percentages (%), processed by chi-square (χ^2^) test. *P* < 0.05 was considered to indicate a statistically significant difference.

## Results

### Participants

Fifty-seven Patients completed the study. Ten participants from the exercise group (two occurred peritonitis and eight because of poor adherence) and three patients from the control group (due to catheter infection) dropped out before the 12-week visit (Figure 1). Participants' demographic and clinical characteristics are described in Table 1. Sex, age, duration of PD, education, body mass index (BMI), cause of ESRD, and medications were not significantly different between the control and Baduanjin exercise groups (*P* > 0.05).

**Table 1:** Baseline characteristics between Baduanjin exercise and control groups.

	**Baduanjin** **exercise group** **(*n* = 25)**	**Control group** **(*n* = 32)**	***P*-value**
Age, years, median (IQR)	60.0 (51.0, 66.0)	62.0 (54.5, 67.3)	0.535
Duration of PD, months, median (IQR)	60.0 (27.0, 118.0)	35.5 (23.5, 102.0)	0.260
BMI (kg/m^2^), mean ± SD	22.4 ± 3.3	22.9 ± 3.1	0.560
Sex, *n* (%)			0.985
Male	14 (56.0%)	18 (56.3%)	
Female	11 (44.0%)	14 (43.8%)	
Education, *n* (%)			0.578
High school and above	12 (48.0%)	13 (40.6%)	
Middle school and below	13 (52.0%)	19 (59.4%)	
Cause of ESRD, *n* (%)			0.229
Glomerulonephritis	15 (60.0%)	11 (34.4%)	
Hypertension	2 (8.0%)	9 (28.1%)	
Diatebes	2 (8.0%)	2 (6.3%)	
Ig A nephropathy	1 (4.0%)	3 (9.4%)	
Other	5 (20.0%)	7 (21.9%)	
Medications, *n* (%)			
Antihypertensives	12 (48.0%)	20 (62.5%)	0.274
Antidiabetics	6 (24.0%)	8 (25.0%)	0.931
Erythropoietic stimulant	16 (64.0%)	14 (43.8%)	0.129
Antidyslipidemic	9 (36.0%)	9 (28.1%)	0.526

*BMI, body mass index; ESRD, end stage renal disease; PD, peritoneal dialysis; IQR, interquartile range; SD, standard deviation*.

*^†^Denotes a 2-sided Fisher exact test*.

### Physical Function

**Figure 3:**
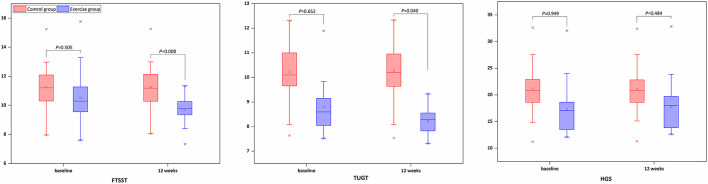
Comparison of baseline and follow-up physical function.

**Figure 4:**
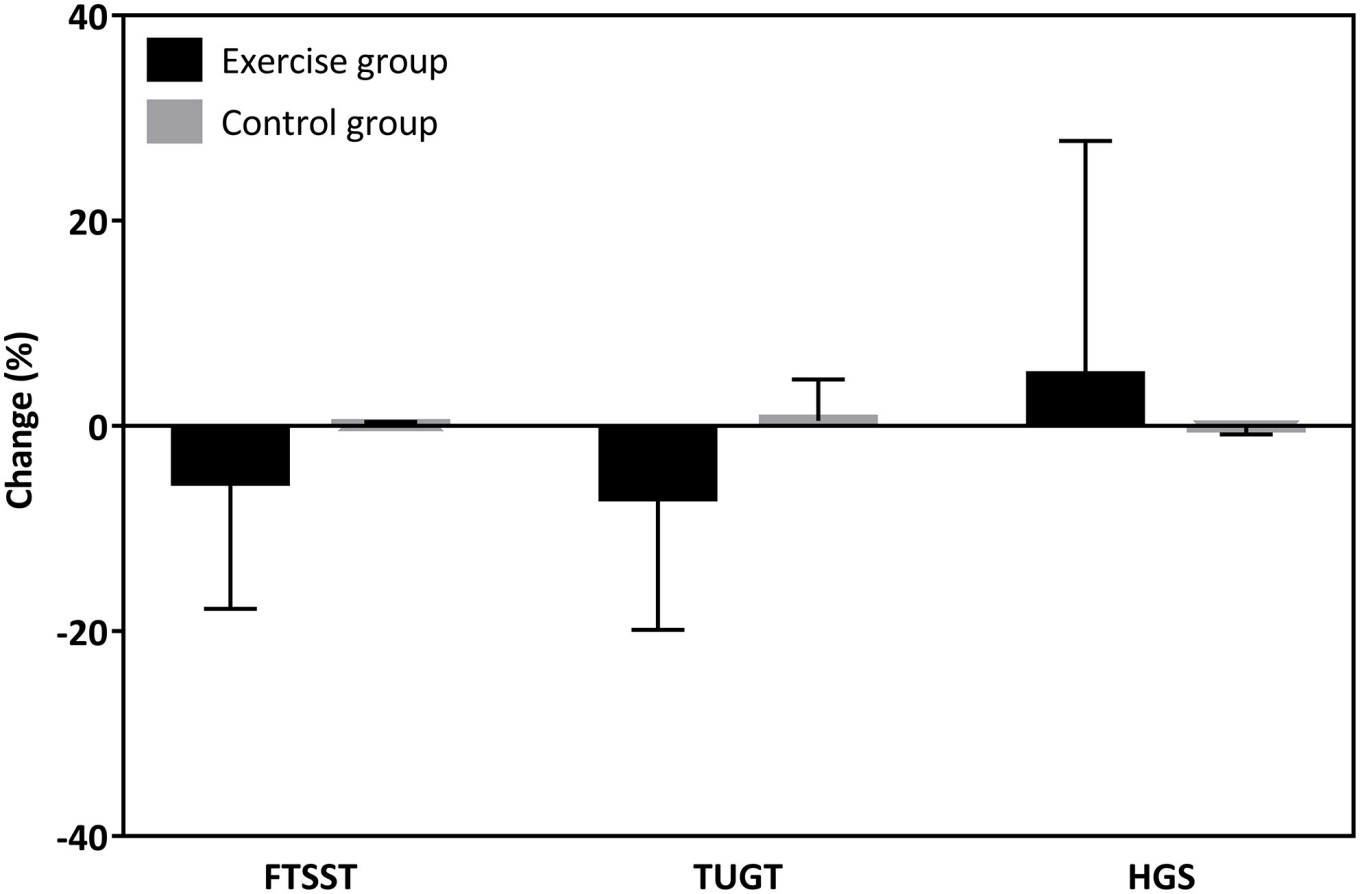
Percentage changes in physical function of the Baduanjin exercise and the control groups.

There was no significant difference for FSTTS, TUGT, and HGS between the Baduanjin exercise and control groups in terms of baseline. The intervention group showed a marginally significant improvement in the FTSST (*P* = 0.008) and TUGT (*P* = 0.040), but no statistically significant difference in HGS (*P* = 0.484) over the 12 weeks compared to the control group (Figure 3). From the rate of change, the exercise group only decreases the time to perform FTSST by 5.27 ± 12.56% and perform TUGT by 6.78 ± 13.10%, increasing HGS by 4.74 ± 23.05%. At the same time, the percent change in the FTSST, TUGT, and HGS in the control group were 0.05 ± 0.36%, 0.48 ± 4.05%, and −0.07 ± 0.77%, respectively (Figure 4).

### Health-Related Quality of Life

There was no significant difference for HRQOL between the Baduanjin exercise and control groups at baseline. Although there was the only effect of kidney disease significantly improved within the Baduanjin exercise group (*P* = 0.02; Table 2), it was observed that HRQOL significantly improved in the fields of the physical component score, mental component score, and effect of kidney disease in comparison to control group following Baduanjin exercise program (*P* < 0.01; Table 2).

**Table 2:** Comparison of HRQOL between two groups.

	**Exercise group (*****n*** **= 25)**				**Control (*****n*** **= 32)**				***P* ^**#**^**
	**Baseline**	**12 weeks**	***Z***	P^*^	**Baseline**	**12 weeks**	***Z***	P^*^	
PCS	41.7 (35.4, 75.0)	58.3 (45.8, 70.8)	−1.172	0.241	39.6 (17.7, 57.3)	40.0 (26.1, 46.5)	−0.290	0.772	<0.001
MCS	65.0 (42.9, 75.4)	69.2 (54.6, 74.6)	−0.592	0.554	65.4 (39.8, 78.4)	54.2 (45.7, 60.0)	−1.216	0.224	<0.001
BKD	37.5 (18.8, 56.3)	37.5 (28.8, 46.9)	−0.029	0.977	43.8 (18.8, 56.3)	37.5 (31.3, 43.8)	−0.587	0.557	0.673
S/P	77.1 (65.7, 81.3)	77.1 (70.9, 81.3)	−1.252	0.211	75.0 (62.5, 83.3)	75.0 (70.8, 79.2)	−0.854	0.393	0.309
EKD	62.5 (51.6, 68.8)	68.8 (56.3, 71.9)	−2.315	0.021	62.5 (47.7, 75.0)	56.3 (44.6, 62.5)	−1.106	0.269	<0.001

*PCS, physical component score; MCS, mental component score; BKD, burden of kidney disease; S/P, symptom/problem; EKD, effect of kidney disease*.

*Data are represented as median with the interquartile range*.

* *Comparison of HRQOL at baseline and 12 weeks within groups*.

# *Comparison of HRQOL at 12 weeks between the Baduanjin exercise and the control groups*.

## Discussion

To our knowledge, this is the first randomized trial of the Baduanjin exercise program in PD patients, so direct comparison with prior studies is limited. Our study suggests that a 12-week Baduanjin exercise program may improve physical function and HRQOL in PD patients. This finding is consistent with results published by Bennet et al. (20), who demonstrated a statistically significant improvement in physical mobility, measured by TUGT, in PD patients who followed a combined resistance and aerobic exercise program.

The poor physical function puts the patients at risk of impaired HRQOL and higher mortality among PD patients linked to a more sedentary lifestyle (4, 21). Regular exercise programs or encouraging increased physical activity may improve the prognosis of PD patients (22). Consistent evidence shows that the Baduanjin exercise program improved physical function (e.g., balance ability, cardiopulmonary fitness, and functional mobility) in Parkinson's disease patients (23). Our results complement prior researches on exercise programs on physical function in PD patients. This study showed an increase in FTSST by 5.27% and TUGT by 6.78% in the Baduanjin exercise group. The findings are somewhat similar to Uchiyama et al. (24) but are slightly lower than Lo's study (25). In previous reports, Uchiyama et al. (24) conducted a randomized controlled trial of 24 PD patients, and results showed that 12-week home-based aerobic exercise improved incremental shuttle walking test, an indicator that assesses mobility, compared to the control group (24). Similarly, another study of 13 PD patients demonstrated that aerobic capacity increased by 16.2% after a 12-week exercise program (25).

In the present study, HRQOL was assessed using the KDQOL-SF. The scores that we recorded at baseline before the intervention are similar to those reported by Hiramatsu et al. (26) in a sample of PD patients. Of particular note is the observation that our 12-week intervention led to a mean increase of FTSST and TUGT in the Baduanjin exercise group and HRQOL with a corresponding change in both. Increased muscle strength and functional mobility may significantly impact HRQOL as daily activities require submaximal efforts. Meanwhile, we observed there was a trend of deterioration in HRQOL in the control group. As everyone knows, PD patients have a more severe disease burden and progressively worsen over time, making their HRQOL unpleasant. Maintenance even increases in physical function are considered as a key to improving HRQOL (27). Our results illustrate further this view.

Several limitations of the study should be noted. Firstly, the rate of lost participants in the current study was relatively high, which might impact the reliability of results to a certain extent. Secondly, among exercise programs, this study lacks precise tools (e.g., accelerometer) to monitor the exercise intensity of patients, although Baduanjin is low-to-moderate intensity. Thirdly, despite our results illustrating that Baduanjin shows at least short-term physical function benefits, studies of even longer duration are required to clarify the role of Baduanjin in the prognosis of PD patients.

## Conclusion

In summary, a 12-week Baduanjin exercise program effectively improved physical function and improved some aspects of HRQOL among PD patients. Larger studies and longer follow-up are needed to determine if these findings will decrease the risk of disease progression.

## Data Availability Statement

The raw data supporting the conclusions of this article will be made available by the authors, without undue reservation.

## Ethics Statement

The studies involving human participants were reviewed and approved by Ethics Committee of Longhua Hospital Shanghai University Traditional Chinese Medicine. The patients/participants provided their written informed consent to participate in this study.

## Author Contributions

QYS, LYH, and JL: research idea and study design. JL, WHZ, and HW: patient supervision and data acquisition. FZ: data analysis. FZ and HCZ: writing a draft and revising. All authors contributed to the article and approved the submitted version.

## Funding

This study was supported by Longhua Hospital Shanghai University Traditional Chinese Medicine (Y1829 and Y21026).

## Conflict of Interest

The authors declare that the research was conducted in the absence of any commercial or financial relationships that could be construed as a potential conflict of interest.

## Publisher's Note

All claims expressed in this article are solely those of the authors and do not necessarily represent those of their affiliated organizations, or those of the publisher, the editors and the reviewers. Any product that may be evaluated in this article, or claim that may be made by its manufacturer, is not guaranteed or endorsed by the publisher.
